# Coinfection and clinical impact of enterotoxigenic *Escherichia coli* harboring diverse toxin variants and colonization factors: 2017-2022

**DOI:** 10.1016/j.ijid.2024.107365

**Published:** 2024-12-16

**Authors:** Mohammad Ashraful Amin, Marjahan Akhtar, Zahid Hasan Khan, Md Taufiqul Islam, Md. Golam Firoj, Yasmin Ara Begum, Sadia Isfat Ara Rahman, Mokibul Hassan Afrad, Taufiqur Rahman Bhuiyan, Fahima Chowdhury, A.S.G. Faruque, Edward T Ryan, Firdausi Qadri, Ashraful Islam Khan

**Affiliations:** 1 Infectious Diseases Division, International Centre for Diarrhoeal Disease Research Bangladesh (icddr,b), Dhaka, Bangladesh; 2 Division of Infectious Diseases, Massachusetts General Hospital, Boston, Massachusetts, USA; 3 Department of Medicine, Harvard Medical School, Boston, Massachusetts, USA; 4 Department of Immunology and Infectious Diseases, Harvard T.H. Chan School of Public Health, Boston, Massachusetts, USA

**Keywords:** Co-infections, clinical impact, diarrheal diseases, enteric pathogens, enterotoxigenic e. coli (ETEC)

## Abstract

**Objectives:**

The severity of the diarrhea disease is exacerbated by co-infections that involve Enterotoxigenic *Escherichia coli* (ETEC) and other enteric pathogens, which complicate the diagnosis and treatment. This study explores the prevalence, clinical manifestations, and risk factors of ETEC and its co-infections in Dhaka, Bangladesh.

**Methods:**

The study used data from the Diarrheal Disease Surveillance System at Dhaka Hospital, involving 16,276 patients from 2017 to 2022. We identified 1404 ETEC-positive patients from the 16,276 data points to investigate the association between ETEC infections, co-infections, and clinical outcomes.

**Results:**

ETEC was identified in 1404 (8.6%) of cases, representing the most common infection among adults (56.6%). ETEC co-infection occurred combined with *V. cholerae* (23%), *Aeromonas* (14.6%), rotavirus (11.8%), *Campylobacter* (6.5%), and *Shigella* spp. (1.7%), respectively. Adults were more likely to develop co-infections with ETEC and *V. cholerae*, while children under five were more likely to develop ETEC co-infections with rotavirus. Co-infections with *V. cholerae* , rotavirus, and *Salmonella* spp. increased the likelihood of fever, while ETEC co-infections with *V. cholerae* increased risks of vomiting, dehydration, and intravenous fluids.

**Conclusions:**

ETEC and co-infections exacerbate illness severity and overburden healthcare systems. Policymakers should prioritize resilient healthcare strategies for ETEC and co-infections.

## Introduction

Enterotoxigenic *E. coli* (ETEC) infection is a one of the common causes of acute watery lasting generally for 3-5 days. ETEC is a major contributor to diarrhea in children and adults, particularly in developing countries and among travelers [1]. ETEC is transmitted via the faecal-oral route, with contaminated water or food serving as the primary drivers [2]. ETEC strains induce illness by several pathogenic components including via the actions of heat-labile enterotoxin [3] and/or heat stable enterotoxin (ST), both of which interfere with intestinal functionality resulting in diarrhea that can be both copious and watery in consistency. In comparison to the presence of ETEC expressing LT alone, symptoms are frequently more severe when ST is present (either alone or in combination with LT) [4]. ETEC produces a diverse range of mostly plasmid-encoded adhesion factors such as colonization factors (CFs) that help with the process of colonizing the intestines. In regions with high prevalence of ETEC, 51,186 deaths have been calculated from 1990 to 2016, which is due to diarrhea of different degrees of severity [5].

Since 1990, the incidence of diarrhea has remained elevated, especially in low-income and middle-income countries (LMICs) that have limited access to healthcare, clean water, and sanitation. Additionally, there is a scarcity of suitable healthcare facilities, diagnostics, and therapeutic treatments in these regions [6]. The Bangladesh Multiple Indicators Cluster Survey (MICS) in 2019 revealed that about 82% of households in Bangladesh drank water contaminated with diarrheagenic bacteria such as ETEC, an increase from approximately 62% in 2012-2013, as reported by MICS 2012-13 (Bangladesh Bureau of Statistics and UNICEF Bangladesh 2014). Public health studies, have utilized various methodologies to identify ETEC as the etiology for diagnosis of outbreaks of food-borne and waterborne illnesses in Bangladesh. Furthermore, during the course of the 2% surveillance system that is carried out at icddr,b hospital ETEC has been detected in diarrheal stools with different frequencies generally and in was highest, about 12% in 2022 [7].

Co-infection is documented when stool specimen detects ETEC as well as other enteric pathogens [8]. Coinfections may contribute to higher rates of morbidity and mortality [9]. Enteric coinfections involving multiple pathogens infecting the gastrointestinal tract simultaneously, pose significant challenges in diagnosing, treating, and caring for patients and in identifying the main pathogens for the cause of diarrheal illness. These infections can exacerbate disease severity, prolong symptoms, and complicate clinical outcomes, malnutrition and stunting [10]. This analysis aims to address the lack of data on enteric coinfections (ETEC) in diarrhea patients in high-risk urban areas like Dhaka. It evaluates demographics, hygiene, and exposure to contaminated sources to determine risk factors. The study also examines the association between toxins, colonization factors, and co-infections. The findings will inform public health interventions, treatment protocols, and preventive measures to improve patient outcomes. ETEC can also cause asymptomatic infections, a common issue in developing countries.

## Methods

### Surveillance

Annually, the Dhaka Hospital of icddr,b provides medical care and complimentary treatment to around 200,00 0 individuals suffering from diarrheal diseases [11]. The icddr,b Dhaka hospital’s "diarrheal Disease Surveillance System (DDSS)" collects information on every 50th patient (2% systematic routine surveillance system) based on their hospital ID number from all over Dhaka city and other parts of the country from patients who seek treatment [11]. For this purpose, stools are tested for the purpose of identifying enteric pathogens. All information stored is utilized for research and publication purposes and this 2% surveillance is approved by the IRB of icddr,b. in this surveillance system, a fecal specimen is obtained and sent to the icddr,b laboratory for microbiological assessment (described below).

This study analyzed data from the iccdr,b surveillance system from 2017 to 2022, representing 16,276 clinical encounters. Of these, we identified 1404 ETEC-positive patients. This study utilized data from a 2% surveillance system, encompassing information on sociodemographic factors (age, sex), WASH variables (drinking water source, toilet type), and clinical data, which included dehydration status, duration of diarrhea, presence of fever, abdominal pain, vomiting, length of hospitalization, and rehydration status.

### Determination of etec toxins and colonization factors

Stool and/or rectal swab (RS) samples were obtained from DDSS patients visiting the icddr,b hospital. Standard microbiological approaches are used to identify enteric pathogens [12,13]. In the present analysis, standard microbiological, conventional serotyping, and molecular techniques were used to in addition analyze for ETEC samples in the Mucosal Immunology and Vaccinology Unit at the icddr,b. For detection of ETEC, stool samples/rectal swabs were streaked on MacConkey agar and then left to grow overnight at 37 °C. To assess whether ETEC toxin LT and ST were present, a multiplex PCR method was used, which has been detailed previously [14]. Next, toxin-positive colonies were put on colonization factor antigen (CFA) agar with and without bile salts. Expression of CFs, including CFA/I, CS1, CS2, CS3, CS4, CS5, CS6, CS7, CS8, CS12, CS14, and CS17 were assessed using CF-specific monoclonal antibodies by dot-blot immunoassay [15]. Also, the isolates were grown on Trypticase Soy Agar plates and tested for CS21 in the same way that was described previously [16].

### Identification of the other co-pathogens

Standard microbiological and biochemical techniques were used to other enteric pathogens, such as *V. cholerae,* Shigella, Salmonella, *Campylobacter, Aeromones* species, and rotavirus [17,18]. Stool ‘cultures on MacConkey and Salmonella-Shigella (SS) agar identified Shigella using standard methods [19]. ProSpect kit (R240396, Oxoid) detected group A rotavirus antigen using enzyme immunoassay [20,21]. For *V. cholerae* detection, stool underwent enrichment in broth and Tellurite Taurocholate Gelatin Agar (TTGA), followed by isolation on TTGA media [22]. As part of standard procedure, stool samples are routinely tested for Salmonella and others using selective plates [22].

### Statistical analysis

Descriptive statistics, including frequencies and percentages, were used to summarize demographic and clinical-pathological data. The relationships between factors contributing to ETEC cholera co-infection and clinical symptoms were assessed using Pearson’s Chi-square test and Fisher’s exact test for sparse data. We present proportions and rates of enterotoxin types and CFs and measure the associations with ETEC and other coinfections. Clinical symptoms were assessed with ETEC and coinfections as well as virulence factors (toxin type and CF) using bivariate and multiple logistic regression modeling. Sociodemographic and Water, Sanitation and Hygiene (WASH) related covariates that were significantly associated with *P* value < 0.05 were adjusted in the multiple regression model to assess association with clinical symptoms. All the statistical tests were conducted with two tailed at 5% level of significance. The data was analyzed using the R statistical software, version 4.2.1. Again, we classified the clinical severity using the WHO dehydration status criteria during the analytic phase. The nutritional status for adults were measured using their body mass index (BMI) where, BMI below 18.5 indicates underweight, 18.5 to 24.9 indicates normal and more than or equal 25 indicates over-weight. Children’s nutritional status was assessed using WHO z-scores, based on the WHO Child Growth Standards for children up to 60 months and WHO Growth References for older children. A z-score −1 to −2 SD, −2 to −3 SD and below −3 SD indicated mild, moderate and severe underweight (low weight-for-age), stunting (low height-for-age), and wasting (low weight-for-height) respectively. And more than + 2 SD of weight for height z-score indicates over-weight or obesity.

## Results

ETEC was identified in 1404 out of the 16,276 total samples, representing 8.6% of cases Adults accounted for 795 cases (56.6%), followed by children under 5 years old with 548 cases (39%). ETEC infections were more common in males, with 772 cases (8.2%), compared to females, with 632 cases (9.3%) (Table 1).

### ETEC and V. cholerae co-infection

The number of only ETEC cases was 628 (48.2%). Notable findings revealed that coinfections with ETEC were common, particularly with *V. cholerae,* with 308 cases (21.9%) observed in comparison to all ETEC positive cases 248 (80.5%) of co-infections of ETEC and *V. cholerae* were in individuals ≥18 years and 53.6% were in males (Table 1).

### ETEC with other pathogens

The number of any co-infections with ETEC was 776 (55.2%). ETEC co-infection occurred alongside *V. cholerae* (23%), *Aeromonas* (14.6%), rotavirus (11.8%), *Campylobacter* (6.5%), and *Shigella* spp. (1.7%), respectively. *Plesiomonas shigelloides* showed relatively lower co-infection rates (0.1%). In contrast, ETEC co-infections with rotavirus, Shigella and *Aeromonas* were more common in both males and females (Table 1). Again, 34 cases were identified in which individuals had co-infections involving, as well as more than one additional enteric bacteria and rotavirus. The specific combinations observed included ETEC with rotavirus and Shigella in one case, ETEC with rotavirus and *Campylobacter* in nine cases, ETEC with rotavirus and *V. cholerae* in eight cases, and ETEC with rotavirus and *Aeromonas* in 16 cases (Supplementary Table 1). The data indicate that co-infections involving ETEC with rotavirus and *Aeromonas* are most prevalent in children under 5 years old, with males being more affected overall. There are minimal co-infections in ≥18 years age groups (Supplementary Table 1).

### Risk factor associated with etec and also etec with coinfection

ETEC without coinfection was 23% (OR: 0.77, CI: 0.62-0.96) and 57% (OR: 0.43, CI: 0.24-0.77) less likely to occur in ≥18 years and 5-17 years of age group respectively compared to the <5 years of age group. ETEC and Rotavirus coinfection was 96% (OR: 0.04, CI: 0.02, 0.08) less likely to occur in ≥18-year-old age group compared to under 5 years of age group. Again, the odds of the coinfection of ETEC and *V. cholerae* in 5-17 years age group was 10.12 (OR: 10.12; CI: 5.47,18.72) times higher than the odds of that in under 5 years of age group. Similarly, it was 6.57 (OR: 6.57; CI: 4.52-9.56) times higher in ≥18 years of age groups people compared to the children under 5 years old (Table 2).

### ETEC related clinical symptoms and wash factors to outcomes

We found significant associations in the clinical symptoms with demographic characteristics and WASH behavior among the ETEC patients. Children under 5 years of age with ETEC were more prone (57%) to suffer from diarrhea that lasted for more than 24 h and required a hospital stay of more than one day (7% among children under 5). They were also more likely to have a fever (55% among younger children). Those in the 5-17 years age range were more likely to report abdominal pain (68% among 5-17 years children), vomiting (92% among 5-17 years children) and dehydration (94% among adults) and required rehydration with IV fluids (70% among older children and 69% in adults). Females suffered from abdominal pain (65% vs 56% among male *P* = 0.002) and needed rehydration IV (50% whereas 43% in male) more than the male patients. Patients who used unsafe sources except for safe drinking water were more susceptible to vomiting (83% vs 73% who used safe water; *P* ≤ 0.001) and become dehydrated (73% vs 64% among safe water users; *P* ≤ 0.001) and may more required to use rehydration IV (49% whereas 43% in safe water users’ patients; *P* = 0.027). Patients who used nonsanitary types of toilet suffered more from abdominal pain which was about 73% compare to the sanitary toilet users (59%) (Table 3).

In multiple logistic regression model analysis, we found that ETEC coinfection with rotavirus and Salmonella *spp.* was associated with 1.8 and 3.2 times respectively more likely to report fever than other ETEC infections. ETEC without any coinfection had 1.53 and 1.27 times much more likely to have diarrhea for more than or equal to 24 h and to have abdominal pain respectively. ETEC with *V. cholerae* had 2.85, 6.21 and 6.04 times much more likely to have vomiting, dehydration and requirement of rehydration and intravenous fluid respectively whereas they were less likely to have fever, abdominal pain and diarrhea for more than 24 h (Table 4).

### Toxin and cf distribution

CFs and toxin types have been associated with ETEC infection and coinfections. All of the toxins (LT, ST, and LT/ST) had a significant association (*P* < 0.001) to CF in patients who only had ETEC (53% of patients), ETEC with *Aeromones* (18%, *P* = 0.030), and *Campylobacter* (10%, *P* < 0.001). Again, the presence of any toxin in stool specimens was significantly associated (*P* < 0.001 and *P* = 0.002 respectively) with *V. cholerae* (29%) and *Salmonella spp.* (2%) in CF negative patients. In LT positive patients, CF positive (17%) had higher Campylobacter that those that were CF negative (6%). Even among (ST) patients, only ETEC (61%) and ETEC with *Campylobacter* (9%) were higher among CF positive than CF negative (39% and 4%, respectively), but ETEC with *V. cholerae* coinfection was higher among CF negative (31%). Finally, among LT/ST, CF-positive patients had higher ETEC without coinfection (51%) and ETEC with *Aeromones* (25%) than CF negative patients (38% and 14%), while *V. cholerae* was higher in CF-negative ETEC patients (35% vs 8%) (Table 5). Those with CF ETEC positive diarrhea were 1.7 times more likely to have abdominal pain than the CF negative patients (Supplementary Table 02). In co-infections with ETEC, colonization factors (CF) such as CFA/I and CS6 were common. Among all toxin type, different types of CF showed a significant association with only ETEC (*P* < 0.001), ETEC with *V. cholerae* (*P* < 0.001), and ETEC with Campylobacter (*P* < 0.001). For LT toxin, there was a significant association between CF types and Campylobacter (*P* = 0.029). ST toxin type patients showed significant association between CF types and only ETEC (*P* < 0.001) and ETEC with *V. cholerae* (*P* < 0.001) respectively. For LT/ST toxin, significant associations were found with only ETEC (*P* = 0.002), ETEC with rotavirus (*P* = 0.029), ETEC with *V. cholerae* (*P* < 0.001), and ETEC coinfections with Aeromonas (*P* = 0.017) (Supplementary Table 03). A significant association was found between age and toxin type (LT, ST, SL/ST) (*P* < 0.001), while gender and WASH factors (drinking water, toilet) had no association with toxin type (Supplementary Table 04).

### Nutritional status with coinfection

A significant association had found between ETEC without any co-infection and nutritional status among the adult patients. For children, in the assessment of nutritional status based on weight-for-age z-scores, ETEC with *V. cholerae* showed a significant association (<0.001). Severe underweight was most prevalent in children with only ETEC infection (38%), followed by those with ETEC co-infections with rotavirus and *V. cholerae* (both at 24%). Regarding height-for-age z-scores, a significant association was noted for ETEC with *V. cholerae* co-infection (*P* = 0.001), with 18% of children exhibiting severe stunting. For weight-for-height z-scores, wasting was highest in cases of only ETEC (48%) followed by ETEC with rotavirus co-infection (22%) and demonstrated a significant association with *V. cholerae* (*P* < 0.001) (Supplementary Table 5).

## Discussion

Co-infections of ETEC with other enteric pathogens, particularly *V. cholerae,* were common and had a significant impact on disease severity. These co-infections were linked to more severe clinical outcomes, including increased vomiting, dehydration, and the need for intravenous rehydration. Specifically, co-infections with rotavirus and *Salmonella* spp. were associated with a higher likelihood of fever, while co-infection with *V. cholerae* exacerbated symptoms such as vomiting, dehydration, and the need for rehydration. Additionally, CF-positive ETEC infections were correlated with more severe symptoms, including abdominal pain and prolonged diarrhea.

The prevalence of enterotoxigenic *E. coli* (ETEC) isolated from stool samples collected from hospitalized patients was found to be 8.6%. This rate is slightly higher than the 8.2% prevalence previously reported in a study of hospitalized children under five years of age in Jakarta [23]. Heat stable (ST) toxin was predominant in ETEC with *Aeromonas* coinfection (11%), while (17%) equal produced heat labile toxin [3] by both ETEC coinfection with rotavirus and *Campylobacter*. Strains with both LT and ST toxins were highest (18%) with ETEC coinfection with *Aeromones.* A previous study in Jakarta found that 72% of the ETEC isolates produced only the heatstable toxin (ST), while 23% produced only the heat-labile toxin (LT). [24]. In a study conducted in the USA [25], both ST and LT enterotoxin genes were detected in 28% of ETEC confirmed cases, ST alone in 54%, and LT alone in 19%. Our study, however, found that 53% of ETEC cases had both ST and LT enterotoxin genes, 39% had ST alone, and 43% had LT alone. We also observed that ETEC infections affected people of all ages, with the highest proportion of cases occurring in children under five years old. These children were often co-infected with rotavirus, Shigella, and *Campylobacter*. In the case of adults, ETEC coinfection with *V. cholerae,* Salmonella, *Aeromonas,* and *Plesiomonas shigelloides* was more prominent. The study found that diarrhea caused by ETEC can affect people of all ages, but it was more commonly isolated from patients over the age of one [26]. Children with diarrhea were studied in South Africa [27], and 59% of their stool samples had multiple enteric pathogen combinations. Of these, 53% had two pathogens, 22% had three pathogens, and 25% had four or more pathogens. In our study, 64% had ETEC with one pathogen, slightly higher than in South Africa, while only 2% had ETEC with two pathogens.

Multiple strains of *E. coli* have developed toxins that cause severe intestinal inflammation and pathological damage. These toxins significantly contribute to the virulence and clinical severity of *E. coli* infections. ETEC bacteria secrete toxins that stimulate excessive secretion of electrolytes and water in the gastrointestinal tract. Our study revealed that individuals who were infected with both ETEC and *V. cholerae* had a six-fold higher risk of developing dehydration compared to those who were only infected with ETEC. The frequent occurrence of multiple infections, particularly involving *V. cholerae, Aeromonas* species, and *Campylobacter* species, underscores the intricate challenges in managing diarrheal diseases in the studied population. Studies in Peru [28] also stated, a significant proportion of ETEC infections exhibited co-infection with other enteropathogens, predominantly rotavirus. The observed demographic variation indicates that individuals of different ages may have varying levels of vulnerability to specific combinations of pathogens. This emphasizes the need for customized interventions that are specifically designed for different age groups.

Enterotoxigenic *E. coli* and coinfections were also related to the type of toxins. LT and ST were significantly associated with ETEC and coinfections with *V. cholerae,* Salmonella, *Aeromones,* and *Campylobacter*. Our findings also indicated that LT was also associated with ETEC and *Campylobacter* coinfection, while ST was associated with ETEC and *V. cholerae* and *Campylobacter* coinfections. ETEC coinfection with various pathogens (except rotavirus and Shigella) was significantly associated with the presence of specific colonization factors (CF). ETEC and coinfections appeared to depend on virulence factors and CF. This differs from previous reports. ccording to López-Vidal et al. [29] in an epidemiological study in urban Mexico, CFA/I, CFA/II, and CFA/IV was not associated with an increased risk of acute diarrhea. However, Cravioto et al. observed that ETEC strains producing colonization factors (CFs) were more likely to cause diarrhea in rural Mexico. In contrast, Paniagua et al. found that only the CFA/I colonization factor was associated with disease in Nicaragua [30]. The study also investigates the associated between toxins (LT and ST) and colonization factors (CFs) in ETEC and coinfections. The analysis uncovers connections between distinct toxins and simultaneous infections with *V. cholerae,* Salmonella, *Aeromonas, and Campylobacter.*

One of the primary strengths of the study was its use of a systematic, large and unbiased sampling surveillance system. In addition, the study addressed the often-overlooked issue of co-infections, providing valuable insights into their impact on disease severity and outcomes. There are several limitations for this analysis. Firstly, our analyses did not consider the specific measurements obtained from the TaqMan polymerase chain reaction tests for each individual pathogen. In future modeling studies that use quantitative CT data, it will be necessary to clearly define the assumptions that differentiate between active infections and subclinical carriage, particularly in asymptomatic stools. Secondly, numerous pathogens such as noroviruses, EAEC, Salmonella, or *Campylobacter* have the ability to inhabit the intestine and can be detected in feces for several weeks even after the disappearance of clinical symptoms. Finally, there is an urgent requirement for research investigating the influence of significant co-infections on the extent of diarrhea in early childhood, without depending on ecological methods and with the long term follow up of the consequences.

This analysis of data from 2017 to 2022, greatly enhances our knowledge of co-infections involving ETEC and other enteric pathogens in Bangladesh. Identifying specific combinations, risk factors, and clinical manifestations forms the basis for focused interventions, highlighting the importance of integrated public health strategies to reduce the impact of enteric coinfections on population health. However, it is important to note that this study did not employ the TaqMan Array Card method, which limits the identifying co-infections. Use of a qPCR method would more beneficial as it would help with the quantification of burden of pathogens to help determine attributable fraction. Future research incorporating TAC will allow for a more detailed analysis of co-infections, providing valuable insights into the complex relationships between pathogens, demographic disparities, and clinical outcomes. Besides, developing and implementing vaccines targeting ETEC and other common co-pathogens, especially for high-risk populations such as children and travelers, should be prioritized.

## Supplementary Material

1

2

3

4

5

## Figures and Tables

**Table 1:** Age wise distribution of coinfection *ETEC* with different enteric organisms in diarrheal cases.

Age:		Total	< 5 years	5-17 years	≥18 years
	All ETEC infection	1404 (8.6%)	548 (39%)	61 (4.3%)	795 (56.6%)
	ETEC coinfection with rotavirus	157 (11.2%)	145 (92.4%)	0 (0%)	12 (7.6%)
	ETEC coinfection with *V. cholerae*	308 (21.9%)	35 (11.4%)	25 (8.1%)	248 (80.5%)
	ETEC coinfection with *Salmonella spp*.	21 (1.5%)	5 (23.8%)	1 (4.7%)	15 (71.4%)
	ETEC coinfection with *Shigella spp*.	24 (1.7%)	14 (58.3%)	1 (4.2%)	9 (37.5%)
	ETEC coinfection with *Aeromonas*	205 (14.6%)	59 (28.8%)	13 (6.3%)	133 (64.9%)
	ETEC coinfection with *Campylobacter*	91 (6.5%)	49 (53.9%)	3 (3.3%)	39 (42.9%)
	ETEC coinfection with *Ples shigelloides*	2 (0.1%)	0 (0%)	0(0%)	2 (100%)
Gender			Male	Female	
	All ETEC infection	1404	772 (8.2%)	632 (9.3%)	
	ETEC coinfection with rotavirus	157 (11.2%)	89 (56.7%)	68 (43.3%)	
	ETEC coinfection with *V. cholerae*	308 (21.9%)	165 (53.6%)	143 (46.4%)	
	ETEC coinfection with *Salmonella spp*.	21 (1.5%)	5 (23.8%)	16 (76.2%)	
	ETEC coinfection with Shigella spp.	24 (1.7%)	12 (50%)	12 (50%)	
	ETEC coinfection with *Aeromonas*	325 (14.6%)	125 (38.5%)	80 (61.5%)	
	ETEC coinfection with *Campylobacter*	91 (6.5%)	44 (48.2%)	47 (51.8%)	
	ETEC coinfection with Plesiomonas shigelloides	2 (0.1%)	0 (0%)	2 (100%)	

**Table 2:** Risk factor associated with ETEC and also ETEC with coinfection.

Risk factor associated with only ETEC							
Factors	Labels	Only ETEC	All other ETEC	Crude OR (95%CI)	*P* value	Adj. OR (95%CI)	*P* value
Age	<5 years	270 (49%)	278 (51%)	ref.	–	–	–
	5-17 years	18 (30%)	43 (70%)	0.43 (0.24, 0.77)	0.004	0.43 (0.24, 0.77)	0.004
	≥18 years	340 (43%)	455 (57%)	0.77 (0.62, 0.96)	0.019	0.77 (0.62, 0.96)	0.019
Sex	Male	350 (45%)	422 (55%)	ref.	–	–	–
	Female	278 (44%)	354 (56%)	0.95 (0.77, 1.17)	0.613	–	–
Drinking water source	Unsafe	377 (45%)	467 (55%)	ref.	–	–	–
	Safe	251 (45%)	309 (55%)	1.01 (0.81, 1.25)	0.955	–	–
Toilet type	Sanitary/semi sanitary	584 (45%)	717 (55%)	ref.	–	–	–
	Others	44 (43%)	59 (57%)	0.92 (0.61, 1.37)	0.670	–	–
Risk factor associated with ETEC and Rota							
Factors	Labels	ETEC and Rota	All other ETEC	Crude OR (95%CI)	*P* value	Adj. OR (95%CI)	*P* value
Age	< 5 years	145 (26%)	403 (74%)	ref.	–	–	–
	5-17 years	0 (0%)	61 (100%)	0 (0, Inf)	0.974	0 (0, Inf)	0.974
	≥18 years	12 (2%)	783 (98%)	0.04 (0.02, 0.08)	< 0.001	0.04 (0.02, 0.08)	<0.001
Sex	Male	89 (12%)	683 (88%)	ref.	–	–	–
	Female	68 (11%)	564 (89%)	0.93 (0.66, 1.29)	0.649	–	–
Drinking water source	Unsafe	83 (10%)	761 (90%)	ref.	–	–	–
	Safe	74 (13%)	486 (87%)	1.4 (1, 1.95)	0.050	1.21 (0.84, 1.74)	0.305
Toilet type	Sanitary/semi sanitary	145 (11%)	1156 (89%)	ref.	–	–	–
	Others	12 (12%)	91 (88%)	1.05 (0.56, 1.97)	0.876	–	–
Risk factor associated with ETEC and Vibrio Cholerae							
Factors	Labels	ETEC and *Vibrio* *Cholerae*	All other ETEC	Crude OR (95%CI)	*P* value	Adj. OR (95%CI)	*P* value
Age	<5 years	35 (6%)	513 (94%)	ref.	–	–	–
	5–17 years	25 (41%)	36 (59%)	10.18 (5.51, 18.82)	<0.001	10.12 (5.47, 18.72)	<0.001
	≥18 years	248 (31%)	547 (69%)	6.65 (4.57, 9.66)	<0.001	6.57 (4.52, 9.56)	<0.001
Sex	Male	165 (21%)	607 (79%)	ref.	–	–	–
	Female	143 (23%)	489 (77%)	1.08 (0.83, 1.39)	0.572	–	–
Drinking water source	Unsafe	200 (24%)	644 (76%)	ref.	–	–	–
	Safe	108 (19%)	452 (81%)	0.77 (0.59, 1.00)	0.051	0.84 (0.64, 1.1)	0.209
Toilet type	Sanitary/semi sanitary	287 (22%)	1014 (78%)	ref.	–	–	–
	Others	21 (20%)	82 (80%)	0.9 (0.55, 1.49)	0.693	–	–
Risk factor associated with ETEC and Others (Salmonella, Shigella, Campylobacter, Aeromones)							
Factors	Labels	ETEC with (Salmonella, Shigella, Campylobacter, Aeromones)	All other ETEC	Crude OR (95%CI)	*P* value	Adj. OR (95%CI)	*P* value
Age	<5 years	127 (23%)	421 (77%)	ref.	–	–	–
	5-17 years	18 (30%)	43 (70%)	1.39 (0.77, 2.49)	0.272	–	–
	≥18 years	196 (25%)	599 (75%)	1.08 (0.84, 1.4)	0.533	–	–
Sex	Male	186 (24%)	586 (76%)	ref.	–	–	–
	Female	155 (25%)	477 (75%)	1.02 (0.8, 1.31)	0.851	–	–
Drinking water source	Unsafe	202 (24%)	642 (76%)	ref.	–	–	–
	Safe	139 (25%)	421 (75%)	1.05 (0.82, 1.35)	0.704	–	–
Toilet type	Sanitary/semi sanitary	315 (24%)	986 (76%)	ref.	–	–	–
	Others	26 (25%)	77 (75%)	1.06 (0.67, 1.68)	0.814	–	–

**Table 3:** Association between risk factors with clinical symptoms in the ETEC and coinfection.

Factor	Labels	Fever	Duration of diarrhea (≥1 day)	Abdominal Pain	Vomiting	Dehydration (Some/Severe)	Hospital Stay (>1 day)	Rehydration IV needed
**Age Group**	<5 years	294 (55%)	302 (57%)	286 (54%)	394 (74%)	166 (31%)	37 (7%)	51 (10%)
	5-17 years	26 (43%)	13 (22%)	41 (68%)	55 (92%)	55 (92%)	2 (3%)	42 (70%)
	≥18 years	267 (34%)	205 (26%)	491 (63%)	626 (81%)	728 (94%)	30 (4%)	538 (69%)
	**p value** *	**<0.001**	**<0.001**	**0.001**	**0.001**	**<0.001**	**0.036**	**<0.001**
**Gender**	Male	314 (42%)	280 (37%)	420 (56%)	584 (78%)	510 (68%)	37 (5%)	323 (43%)
	Female	273 (44%)	240 (39%)	398 (65%)	491 (80%)	439 (71%)	32 (5%)	308 (50%)
	**p value** *	0.410	0.614	**0.002**	0.507	0.237	0.901	**0.012**
**Drinking water source**	Safe	241 (44%)	221 (41%)	342 (63%)	394 (73%)	347 (64%)	32 (6%)	231 (43%)
	Unsafe	346 (42%)	299 (36%)	476 (58%)	681 (83%)	602 (73%)	37 (5%)	400 (49%)
	**p value** *	0.403	0.111	0.062	**<0.001**	**<0.001**	0.258	**0.027**
**Toilet**	Sanitary	539 (43%)	484 (38%)	744 (59%)	994 (79%)	879 (70%)	60 (5%)	581 (46%)
	Others	48 (48%)	36 (36%)	74 (73%)	81 (80%)	70 (69%)	9 (9%)	50 (50%)
	**p-value** *	0.349	0.670	**0.004**	0.801	>0.999	0.092	0.534

* The Chi-squared test and Fisher’s exact test were conducted as required.P values less than 0.05 were bolded and considered significant.

**Table 4:** Association between clinical symptoms with the ETEC and other coinfection.

		Only ETEC	ETEC with rotavirus	ETEC with *V.* *cholera*	ETEC with Salmonella *spp*.	ETEC with Shigella *spp*.	ETEC with *Aeromones*	ETEC with *Campylobacter*
Fever	No	335 (43%)	54 (7%)	222 (29%)	7 (1%)	8 (1%)	115 (15%)	48 (6%)
	Yes	274 (47%)	99 (17%)	78 (13%)	14 (2%)	15 (3%)	85 (14%)	40 (7%)
	*P*-value	0.187	<0.001	<0.001	0.043	0.034	0.938	0.657
	COR	1.16 (0.93, 1.44)	2.72 (1.92, 3.86)	0.38 (0.29, 0.51)	2.69 (1.08, 6.71)	2.52 (1.06, 5.99)	0.98 (0.72, 1.32)	1.11 (0.72, 1.72)
	AOR^$^	1.1 (0.89, 1.38)	1.82 (1.25, 2.65)	0.48 (0.36, 0.64)	3.19 (1.26, 8.06)	2.24 (0.93, 5.42)	1.07 (0.78, 1.46)	0.99 (0.63, 1.54)
Duration of diarrhea (≥1 day)	No	340 (40%)	63 (7%)	239 (28%)	13 (2%)	12 (1%)	141 (17%)	51 (6%)
	Yes	269 (52%)	90 (17%)	61 (12%)	8 (2%)	11 (2%)	59 (11%)	37 (7%)
	*P*-value	<0.001	<0.001	<0.001	>0.999	0.388	0.007	0.429
	COR	1.59 (1.28, 1.98)	2.6 (1.84, 3.66)	0.34 (0.25, 0.46)	1 (0.41, 2.43)	1.5 (0.66, 3.43)	0.64 (0.46, 0.89)	1.19 (0.77, 1.85)
	AOR^$^	1.53 (1.22, 1.93)	1.33 (0.92, 1.94)	0.48 (0.35, 0.66)	1.24 (0.49, 3.12)	1.22 (0.51, 2.9)	0.71 (0.51, 1)	1 (0.63, 1.59)
Abdominal pain	No	227 (42%)	61 (11%)	133 (24%)	8 (1%)	7 (1%)	79 (14%)	45 (8%)
	Yes	381 (47%)	92 (11%)	167 (20%)	13 (2%)	16 (2%)	121 (15%)	43 (5%)
	*P*-value	0.075	>0.999	0.095	0.999	0.397	0.938	0.032
	COR	1.23 (0.98, 1.52)	1.01 (0.71, 1.42)	0.8 (0.61, 1.03)	1.09 (0.45, 2.64)	1.54 (0.63, 3.76)	1.03 (0.76, 1.39)	0.62 (0.4, 0.95)
	AOR^&^	1.27 (1.02, 1.58)	1.29 (0.88, 1.87)	0.68 (0.52, 0.89)	0.86 (0.35, 2.12)	1.64 (0.66, 4.05)	1 (0.73, 1.37)	0.65 (0.42, 1)
Vomiting	No	153 (53%)	37 (13%)	29 (10%)	7 (2%)	8 (3%)	39 (13%)	24 (8%)
	Yes	456 (42%)	116 (11%)	271 (25%)	14 (1%)	15 (1%)	161 (15%)	64 (6%)
	*P*-value	0.002	0.346	<0.001	0.181	0.123	0.575	0.177
	COR	0.66 (0.51, 0.86)	0.83 (0.56, 1.23)	3.03 (2.02, 4.56)	0.53 (0.21, 1.33)	0.5 (0.21, 1.19)	1.13 (0.78, 1.65)	0.7 (0.43, 1.14)
	AOR^#^	0.66 (0.51, 0.87)	1.05 (0.68, 1.61)	2.85 (1.87, 4.34)	0.52 (0.21, 1.33)	0.53 (0.22, 1.27)	1.11 (0.76, 1.63)	0.71 (0.43, 1.17)
Dehydration (Some/Severe)	No	225 (54%)	95 (23%)	14 (3%)	4 (1%)	13 (3%)	44 (11%)	39 (9%)
	Yes	384 (40%)	58 (6%)	286 (30%)	17 (2%)	10 (1%)	156 (16%)	48 (5%)
	*P*-value	<0.001	<0.001	<0.001	0.341	0.010	0.005	0.004
	COR	0.57 (0.45, 0.72)	0.22 (0.15, 0.31)	12.36 (7.12, 21.43)	1.87 (0.63, 5.6)	0.33 (0.14, 0.76)	1.66 (1.16, 2.37)	0.51 (0.33, 0.8)
	AOR^#^	0.52 (0.38, 0.72)	1.06 (0.71, 1.59)	6.21 (3.39, 11.39)	1.3 (0.31, 5.39)	0.34 (0.11, 1.05)	1.39 (0.88, 2.2)	0.59 (0.32, 1.07)
Duration of Hospital stay (>1 day)	No	573 (44%)	143 (11%)	287 (22%)	21 (2%)	22 (2%)	191 (15%)	84 (6%)
	Yes	36 (52%)	10 (14%)	13 (19%)	0 (0%)	1 (1%)	9 (13%)	3 (4%)
	*P*-value	0.215	0.333	0.654	0.621	>0.999	0.861	0.619
	COR	1.37 (0.85, 2.23)	1.37 (0.68, 2.73)	0.82 (0.44, 1.51)	0 (0, Inf)	0.85 (0.11, 6.41)	0.87 (0.42, 1.78)	0.66 (0.2, 2.13)
	AOR^$^	1.32 (0.81, 2.15)	0.98 (0.47, 2.04)	1.03 (0.53, 1.97)	0 (0, Inf)	0.76 (0.1, 5.76)	0.93 (0.45, 1.91)	0.6 (0.18, 1.96)
Rehydration IV needed	No	390 (53%)	137 (19%)	51 (7%)	12 (2%)	17 (2%)	97 (13%)	56 (8%)
	Yes	219 (35%)	16 (3%)	249 (39%)	9 (1%)	6 (1%)	103 (16%)	31 (5%)
	*P*-value	<0.001	<0.001	<0.001	0.828	0.058	0.125	0.045
	COR	0.47 (0.38, 0.58)	0.11 (0.07, 0.19)	8.72 (6.29, 12.08)	0.87 (0.36, 2.08)	0.4 (0.16, 1.03)	1.28 (0.95, 1.73)	0.62 (0.4, 0.98)
	AOR^¥^	0.39 (0.3, 0.52)	0.48 (0.26, 0.87)	6.04 (4.18, 8.73)	0.48 (0.18, 1.29)	0.52 (0.17, 1.56)	1.03 (0.72, 1.48)	0.77 (0.44, 1.34)

Adjusted by ^$^ age; ^&^ age, gender and toilet type; ^#^ age and drinking water source; ^¥^ age, gender and drinking water source.

**Table 5:** Association between virulence factors [Toxin and CF Based on positive and negative] with the ETEC and coinfection.

Toxin Type	CF Status	All ETEC	Only ETEC	ETEC with rotavirus	ETEC with *V.* *cholera*	ETEC with Salmonella *spp.*	ETEC with *Shigella spp.*	ETEC with *Aeromones*	ETEC with *Campylobacter*
**All (LT, ST, LT/ST)**	Positive	471 (100%)	249 (53%)	57 (12%)	39 (8%)	1 (0%)	8 (2%)	83 (18%)	46 (10%)
	Negative	894 (100%)	360 (40%)	96 (11%)	261 (29%)	20 (2%)	15 (2%)	117 (13%)	42 (5%)
	**p-value** ^*^	-	**<0.001**	0.471	**<0.001**	**0.002**	>0.999	**0.030**	**<0.001**
**LT**	Positive	107 (100%)	46 (43%)	18 (17%)	10 (9%)	0 (0%)	2 (2%)	16 (15%)	18 (17%)
	Negative	215 (100%)	100 (47%)	38 (18%)	37 (17%)	2 (1%)	6 (3%)	24 (11%)	12 (6%)
	**p-value** ^*^	-	0.555	>0.999	0.066	>0.999	>0.999	0.371	**0.002**
**ST**	Positive	174 (100%)	107 (61%)	16 (9%)	13 (7%)	1 (1%)	5 (3%)	20 (11%)	16 (9%)
	Negative	307 (100%)	120 (39%)	31 (10%)	94 (31%)	9 (3%)	5 (2%)	42 (14%)	11 (4%)
	**p-value** ^*^	-	**<0.001**	0.873	**<0.001**	0.103	0.507	0.572	**0.013**
**LT/ST**	Positive	190 (100%)	96 (51%)	23 (12%)	16 (8%)	0 (0%)	1 (1%)	47 (25%)	12 (6%)
	Negative	372 (100%)	140 (38%)	27 (7%)	130 (35%)	9 (2%)	4 (1%)	51 (14%)	19 (5%)
	**p-value** ^*^	-	**0.004**	0.061	**<0.001**	**0.032**	0.667	**<0.001**	0.562

* The Chi-squared test and Fisher’s exact test were conducted as required.P values less than 0.05 were bolded and considered significant.

## Data Availability

The dataset evaluated in this study is not publicly accessible because it was taken from a big hospital surveillance with a substantial dataset. The dataset includes personal information about the study participants, including their names, entrance dates, months, and residential areas. While obtaining consent from the patients, it has been guaranteed that their personal information would remain confidential. However, the findings of the study will be made public. Therefore, if the entire dataset is made available in the publication, supplemental files, or a public repository, it will expose all the personal information of the patients, which should remain confidential. Furthermore, it will also reveal other significant information that has not yet been published. Therefore, icddr,b’s policy that the complete data sets should not be included in the publication, supplemental files, or a public repository. However, a portion of the dataset pertaining to this publication is accessible upon request. Interested readers can reach out to Ms. Armana Ahmed (aahmed@icddrb. org) from the Research Administration & Strategy department of icddr,b to inquire about obtaining the data (http://www.icddrb.org/).
