# Supplementary material for: Coinfection and clinical impact of enterotoxigenic Escherichia coli harboring diverse toxin variants and colonization factors: 2017-2022
Source: Int J Infect Dis. Author manuscript; Available in PMC 2025 Feb 5. (PMC11798591; doi:10.1016/j.ijid.2024.107365)
Supplement: 1 [file NIHMS2049916-supplement-1.docx]

**Supplementary Table 01: *ETEC* coinfection with multiple enteric organisms in diarrheal cases**

|  | | Total number of cases | Male | Female | <5 years | 5 to 17 years | ≥18 years |
| --- | --- | --- | --- | --- | --- | --- | --- |
| ETEC | Rota+ *shigella* | 1 | 0 | 1 | 1 | 0 | 0 |
| ETEC | Rota+ *Campylobacter* | 9 | 4 | 5 | 8 | 0 | 1 |
| ETEC | Rota+ *Vibrio cholerae* | 8 | 6 | 2 | 4 | 0 | 4 |
| ETEC | Rota+ *Aeromones* | 16 | 10 | 6 | 16 | 0 | 0 |
| Total = | | 34 | 20 | 14 | 29 | 0 | 5 |
