# Supplementary material for: Coinfection and clinical impact of enterotoxigenic Escherichia coli harboring diverse toxin variants and colonization factors: 2017-2022
Source: Int J Infect Dis. Author manuscript; Available in PMC 2025 Feb 5. (PMC11798591; doi:10.1016/j.ijid.2024.107365)
Supplement: 2 [file NIHMS2049916-supplement-2.docx]

**Supplementary Table 02: Association with clinical symptoms with the Colonization factors (CF)**

| Clinical characteristics | | **CF Negative** | **Single CF** | **Multiple CF** |
| --- | --- | --- | --- | --- |
| Fever | No | 537 (69%) | 119 (15%) | 122 (16%) |
|  | Yes | 357 (61%) | 115 (20%) | 115 (20%) |
|  | p-value | 0.007 | | |
|  | COR | ref. | 1.45 (1.09, 1.94) | 1.42 (1.06, 1.89) |
|  | AOR^$^ | ref. | 1.15 (0.85, 1.55) | 1.31 (0.98, 1.76) |
| Duration of diarrhea (≥1 day) | No | 572 (68%) | 123 (15%) | 150 (18%) |
|  | Yes | 322 (62%) | 111 (21%) | 87 (17%) |
|  | p-value | 0.006 | | |
|  | COR | ref. | 1.6 (1.2, 2.14) | 1.03 (0.77, 1.39) |
|  | AOR^$^ | ref. | 1.12 (0.82, 1.53) | 0.89 (0.65, 1.22) |
| Abdominal pain | No | 371 (68%) | 76 (14%) | 99 (18%) |
|  | Yes | 523 (64%) | 157 (19%) | 138 (17%) |
|  | p-value | 0.039 | | |
|  | COR | ref. | 1.47 (1.08, 1.99) | 0.99 (0.74, 1.32) |
|  | AOR^&^ | ref. | 1.69 (1.23, 2.32) | 1.07 (0.79, 1.43) |
| Vomiting | No | 201 (69%) | 50 (17%) | 39 (13%) |
|  | Yes | 693 (64%) | 184 (17%) | 198 (18%) |
|  | p-value | 0.126 | | |
|  | COR | ref. | 1.07 (0.75, 1.51) | 1.47 (1.01, 2.15) |
|  | AOR^#^ | ref. | 1.21 (0.84, 1.74) | 1.52 (1.04, 2.23) |
| Dehydration (Some/Severe) | No | 241 (58%) | 95 (23%) | 79 (19%) |
|  | Yes | 652 (69%) | 139 (15%) | 158 (17%) |
|  | p-value | <0.001 | | |
|  | COR | ref. | 0.54 (0.4, 0.73) | 0.74 (0.54, 1.01) |
|  | AOR^#^ | ref. | 1.44 (0.98, 2.12) | 1.06 (0.7, 1.6) |
| Duration of Hospital stay (>1 day) | No | 856 (66%) | 216 (17%) | 223 (17%) |
|  | Yes | 37 (54%) | 18 (26%) | 14 (20%) |
|  | p-value | 0.069 | 0.069 | 0.069 |
|  | COR | ref. | 1.93 (1.08, 3.45) | 1.45 (0.77, 2.73) |
|  | AOR^$^ | ref. | 1.66 (0.91, 3.02) | 1.37 (0.73, 2.59) |
| Rehydration IV needed | No | 451 (62%) | 156 (21%) | 126 (17%) |
|  | Yes | 442 (70%) | 78 (12%) | 111 (18%) |
|  | p-value | <0.001 | | |
|  | COR | ref. | 0.51 (0.38, 0.69) | 0.9 (0.67, 1.2) |
|  | AOR^¥^ | ref. | 1.06 (0.72, 1.56) | 1.25 (0.87, 1.78) |

Adjusted by ^$^ age; ^&^ age, gender and toilet type; ^#^ age and drinking water source; ^¥^ age, gender and drinking water source

*[Colonization factors (CF)- Based on single, multiple, none]
