# Supplementary material for: Coinfection and clinical impact of enterotoxigenic Escherichia coli harboring diverse toxin variants and colonization factors: 2017-2022
Source: Int J Infect Dis. Author manuscript; Available in PMC 2025 Feb 5. (PMC11798591; doi:10.1016/j.ijid.2024.107365)
Supplement: 3 [file NIHMS2049916-supplement-3.docx]

**Supplementary Table 03: Association with types of colonization factors (CF) with ETEC-coinfections**

| **Toxin Type** | **CF Type** | **Only ETEC** | **ETEC with Rotavirus** | **ETEC with** ***V. Cholerae*** | **ETEC with *Salmonella*** | **ETEC with *Shigella*** | **ETEC with *Aeromonas*** | **ETEC with Campylobacter** |
| --- | --- | --- | --- | --- | --- | --- | --- | --- |
| All | Negative | 360 (40%) | 96 (11%) | 261 (29%) | 20 (2%) | 15 (2%) | 117 (13%) | 42 (5%) |
|  | CFA/I | 40 (62%) | 5 (8%) | 3 (5%) | 0 (0%) | 2 (3%) | 10 (16%) | 6 (9%) |
|  | CFA/I+CS21 | 14 (61%) | 4 (17%) | 1 (4%) | 0 (0%) | 0 (0%) | 3 (13%) | 2 (9%) |
|  | CS1+CS3 | 6 (86%) | 0 (0%) | 0 (0%) | 0 (0%) | 0 (0%) | 1 (14%) | 0 (0%) |
|  | CS1+CS3+CS21 | 22 (59%) | 4 (11%) | 3 (8%) | 0 (0%) | 2 (5%) | 9 (24%) | 1 (3%) |
|  | CS14 | 13 (46%) | 5 (18%) | 4 (14%) | 0 (0%) | 0 (0%) | 6 (21%) | 0 (0%) |
|  | CS17 | 15 (44%) | 8 (24%) | 4 (12%) | 0 (0%) | 0 (0%) | 4 (12%) | 4 (12%) |
|  | CS17, CS6 | 1 (100%) | 0 (0%) | 0 (0%) | 0 (0%) | 0 (0%) | 0 (0%) | 0 (0%) |
|  | CS2+CS3 | 19 (76%) | 2 (8%) | 2 (8%) | 0 (0%) | 0 (0%) | 1 (4%) | 0 (0%) |
|  | CS2+CS3, CFA/I+CS21 | 1 (100%) | 0 (0%) | 0 (0%) | 0 (0%) | 0 (0%) | 0 (0%) | 0 (0%) |
|  | CS2+CS3+CS21 | 3 (33%) | 2 (22%) | 2 (22%) | 0 (0%) | 0 (0%) | 1 (11%) | 3 (33%) |
|  | CS21 | 8 (62%) | 0 (0%) | 2 (15%) | 0 (0%) | 0 (0%) | 2 (15%) | 1 (8%) |
|  | CS4 | 1 (33%) | 0 (0%) | 0 (0%) | 0 (0%) | 1 (33%) | 0 (0%) | 1 (33%) |
|  | CS4+CS6 | 3 (38%) | 0 (0%) | 3 (38%) | 0 (0%) | 0 (0%) | 2 (25%) | 0 (0%) |
|  | CS5+CS6 | 55 (48%) | 10 (9%) | 7 (6%) | 0 (0%) | 2 (2%) | 29 (25%) | 13 (11%) |
|  | CS6 | 27 (50%) | 7 (13%) | 2 (4%) | 1 (2%) | 1 (2%) | 9 (17%) | 8 (15%) |
|  | CS6,CS14 | 1 (50%) | 0 (0%) | 0 (0%) | 0 (0%) | 0 (0%) | 1 (50%) | 0 (0%) |
|  | CS6+CFA/III(CS8) | 1 (100%) | 0 (0%) | 0 (0%) | 0 (0%) | 0 (0%) | 0 (0%) | 0 (0%) |
|  | CS6+CS8 | 2 (25%) | 2 (25%) | 2 (25%) | 0 (0%) | 0 (0%) | 2 (25%) | 0 (0%) |
|  | CS7 | 13 (52%) | 3 (12%) | 2 (8%) | 0 (0%) | 0 (0%) | 1 (4%) | 6 (24%) |
|  | CS8 | 3 (100%) | 0 (0%) | 0 (0%) | 0 (0%) | 0 (0%) | 0 (0%) | 0 (0%) |
|  | PCFO159 | 1 (10%) | 5 (50%) | 2 (20%) | 0 (0%) | 0 (0%) | 2 (20%) | 1 (10%) |
|  | **p^*^ value** | **<0.001** | 0.119 | **<0.001** | 0.948 | 0.552 | 0.122 | **<0.001** |
| LT | Negative | 100 (47%) | 38 (18%) | 37 (17%) | 2 (1%) | 6 (3%) | 24 (11%) | 12 (6%) |
|  | CFA/I | 0 (0%) | 0 (0%) | 0 (0%) | 0 (0%) | 0 (0%) | 1 (50%) | 1 (50%) |
|  | CFA/I+CS21 | 0 (0%) | 0 (0%) | 0 (0%) | 0 (0%) | 0 (0%) | 1 (100%) | 0 (0%) |
|  | CS1+CS3 | 2 (100%) | 0 (0%) | 0 (0%) | 0 (0%) | 0 (0%) | 0 (0%) | 0 (0%) |
|  | CS1+CS3+CS21 | 1 (25%) | 1 (25%) | 0 (0%) | 0 (0%) | 2 (50%) | 0 (0%) | 1 (25%) |
|  | CS14 | 0 (0%) | 0 (0%) | 0 (0%) | 0 (0%) | 0 (0%) | 1 (100%) | 0 (0%) |
|  | CS17 | 12 (40%) | 8 (27%) | 3 (10%) | 0 (0%) | 0 (0%) | 4 (13%) | 4 (13%) |
|  | CS2+CS3 | 5 (100%) | 0 (0%) | 0 (0%) | 0 (0%) | 0 (0%) | 0 (0%) | 0 (0%) |
|  | CS2+CS3+CS21 | 1 (50%) | 0 (0%) | 0 (0%) | 0 (0%) | 0 (0%) | 1 (50%) | 0 (0%) |
|  | CS5+CS6 | 5 (38%) | 2 (15%) | 0 (0%) | 0 (0%) | 0 (0%) | 3 (23%) | 3 (23%) |
|  | CS6 | 4 (33%) | 2 (17%) | 2 (17%) | 0 (0%) | 0 (0%) | 2 (17%) | 3 (25%) |
|  | CS6+CFA/III(CS8) | 1 (100%) | 0 (0%) | 0 (0%) | 0 (0%) | 0 (0%) | 0 (0%) | 0 (0%) |
|  | CS6+CS8 | 2 (25%) | 2 (25%) | 2 (25%) | 0 (0%) | 0 (0%) | 2 (25%) | 0 (0%) |
|  | CS7 | 12 (50%) | 3 (12%) | 2 (8%) | 0 (0%) | 0 (0%) | 1 (4%) | 6 (25%) |
|  | CS8 | 1 (100%) | 0 (0%) | 0 (0%) | 0 (0%) | 0 (0%) | 0 (0%) | 0 (0%) |
|  | PCFO159 | 0 (0%) | 0 (0%) | 1 (100%) | 0 (0%) | 0 (0%) | 0 (0%) | 0 (0%) |
|  | **p^*^ value** | 0.190 | 0.973 | 0.632 | >0.999 | 0.159 | **0.073** | **0.029** |
| ST | Negative | 120 (39%) | 31 (10%) | 94 (31%) | 9 (3%) | 5 (2%) | 42 (14%) | 11 (4%) |
|  | CFA/I | 37 (67%) | 4 (7%) | 3 (5%) | 0 (0%) | 2 (4%) | 6 (11%) | 5 (9%) |
|  | CFA/I+CS21 | 14 (67%) | 4 (19%) | 0 (0%) | 0 (0%) | 0 (0%) | 2 (10%) | 2 (10%) |
|  | CS14 | 5 (56%) | 1 (11%) | 1 (11%) | 0 (0%) | 0 (0%) | 2 (22%) | 0 (0%) |
|  | CS17 | 1 (50%) | 0 (0%) | 1 (50%) | 0 (0%) | 0 (0%) | 0 (0%) | 0 (0%) |
|  | CS2+CS3 | 3 (75%) | 0 (0%) | 1 (25%) | 0 (0%) | 0 (0%) | 0 (0%) | 0 (0%) |
|  | CS21 | 7 (70%) | 0 (0%) | 1 (10%) | 0 (0%) | 0 (0%) | 1 (10%) | 1 (10%) |
|  | CS4 | 1 (33%) | 0 (0%) | 0 (0%) | 0 (0%) | 1 (33%) | 0 (0%) | 1 (33%) |
|  | CS4+CS6 | 3 (43%) | 0 (0%) | 2 (29%) | 0 (0%) | 0 (0%) | 2 (29%) | 0 (0%) |
|  | CS5+CS6 | 14 (64%) | 2 (9%) | 4 (18%) | 0 (0%) | 1 (5%) | 0 (0%) | 2 (9%) |
|  | CS6 | 20 (51%) | 5 (13%) | 0 (0%) | 1 (3%) | 1 (3%) | 7 (18%) | 5 (13%) |
|  | CS8 | 2 (100%) | 0 (0%) | 0 (0%) | 0 (0%) | 0 (0%) | 0 (0%) | 0 (0%) |
|  | **p^*^ value** | **<0.001** | 0.911 | **<0.001** | 0.926 | 0.259 | 0.589 | 0.100 |
| LT/ST | Negative | 140 (38%) | 27 (7%) | 130 (35%) | 9 (2%) | 4 (1%) | 51 (14%) | 19 (5%) |
|  | CFA/I | 3 (43%) | 1 (14%) | 0 (0%) | 0 (0%) | 0 (0%) | 3 (43%) | 0 (0%) |
|  | CFA/I+CS21 | 0 (0%) | 0 (0%) | 1 (100%) | 0 (0%) | 0 (0%) | 0 (0%) | 0 (0%) |
|  | CS1+CS3 | 4 (80%) | 0 (0%) | 0 (0%) | 0 (0%) | 0 (0%) | 1 (20%) | 0 (0%) |
|  | CS1+CS3+CS21 | 21 (64%) | 3 (9%) | 3 (9%) | 0 (0%) | 0 (0%) | 9 (27%) | 0 (0%) |
|  | CS14 | 8 (44%) | 4 (22%) | 3 (17%) | 0 (0%) | 0 (0%) | 3 (17%) | 0 (0%) |
|  | CS17 | 2 (100%) | 0 (0%) | 0 (0%) | 0 (0%) | 0 (0%) | 0 (0%) | 0 (0%) |
|  | CS17, CS6 | 1 (100%) | 0 (0%) | 0 (0%) | 0 (0%) | 0 (0%) | 0 (0%) | 0 (0%) |
|  | CS2+CS3 | 11 (69%) | 2 (12%) | 1 (6%) | 0 (0%) | 0 (0%) | 1 (6%) | 0 (0%) |
|  | CS2+CS3, CFA/I+CS21 | 1 (100%) | 0 (0%) | 0 (0%) | 0 (0%) | 0 (0%) | 0 (0%) | 0 (0%) |
|  | CS2+CS3+CS21 | 2 (29%) | 2 (29%) | 2 (29%) | 0 (0%) | 0 (0%) | 0 (0%) | 3 (43%) |
|  | CS21 | 1 (33%) | 0 (0%) | 1 (33%) | 0 (0%) | 0 (0%) | 1 (33%) | 0 (0%) |
|  | CS4+CS6 | 0 (0%) | 0 (0%) | 1 (100%) | 0 (0%) | 0 (0%) | 0 (0%) | 0 (0%) |
|  | CS5+CS6 | 36 (45%) | 6 (8%) | 3 (4%) | 0 (0%) | 1 (1%) | 26 (32%) | 8 (10%) |
|  | CS6 | 3 (100%) | 0 (0%) | 0 (0%) | 0 (0%) | 0 (0%) | 0 (0%) | 0 (0%) |
|  | CS6,CS14 | 1 (50%) | 0 (0%) | 0 (0%) | 0 (0%) | 0 (0%) | 1 (50%) | 0 (0%) |
|  | CS7 | 1 (100%) | 0 (0%) | 0 (0%) | 0 (0%) | 0 (0%) | 0 (0%) | 0 (0%) |
|  | PCFO159 | 1 (11%) | 5 (56%) | 1 (11%) | 0 (0%) | 0 (0%) | 2 (22%) | 1 (11%) |
|  | **p^*^ value** | **0.002** | **0.029** | **<0.001** | 0.844 | >0.999 | **0.017** | 0.145 |

^*^Fisher exact test
