# Supplementary material for: Coinfection and clinical impact of enterotoxigenic Escherichia coli harboring diverse toxin variants and colonization factors: 2017-2022
Source: Int J Infect Dis. Author manuscript; Available in PMC 2025 Feb 5. (PMC11798591; doi:10.1016/j.ijid.2024.107365)
Supplement: 4 [file NIHMS2049916-supplement-4.docx]

**Supplementary Table 04: Association with types of colonization factors (CF) with age and WASH factors**

| **Factor** | **Labels** | **LT** | **ST** | **LT/ST** | **p value** |
| --- | --- | --- | --- | --- | --- |
| **Age Group** | <5 years | 180 (33%) | 165 (30%) | 197 (36%) | <0.001 |
|  | 5-17 years | 11 (18%) | 19 (31%) | 31 (51%) |  |
|  | ≥18 years | 137 (17%) | 385 (49%) | 261 (33%) |  |
| **Gender** | Male | 185 (24%) | 314 (41%) | 264 (35%) | 0.791 |
|  | Female | 143 (23%) | 255 (41%) | 225 (36%) |  |
| **Drinking water source** | Tube well | 203 (24%) | 341 (41%) | 291 (35%) | 0.777 |
|  | Others | 125 (23%) | 228 (41%) | 198 (36%) |  |
| **Toilet** | Sanitary | 304 (24%) | 524 (41%) | 456 (36%) | 0.771 |
|  | Others | 24 (24%) | 45 (44%) | 33 (32%) |  |
