# Supplementary material for: Coinfection and clinical impact of enterotoxigenic Escherichia coli harboring diverse toxin variants and colonization factors: 2017-2022
Source: Int J Infect Dis. Author manuscript; Available in PMC 2025 Feb 5. (PMC11798591; doi:10.1016/j.ijid.2024.107365)
Supplement: 5 [file NIHMS2049916-supplement-5.docx]

**Supplementary Table 05: Association with types of Nutritional status) with ETEC and others coinfection**

| **Nutritional status for adult (≥18 years), N=795** | **Only ETEC** | **ETEC with Rotavirus** | **ETEC with *V. Cholera*** | **ETEC with *Salmonella*** | **ETEC with *Shigella*** | **ETEC with *Aeromonas*** | **ETEC with Campylobacter** |
| --- | --- | --- | --- | --- | --- | --- | --- |
| **BMI, n=600** |  |  |  |  |  |  |  |
| Underweight, n=129 | 44 (34%) | 3 (2%) | 42 (33%) | 2 (2%) | 2 (2%) | 25 (19%) | 13 (10%) |
| Normal, n=363 | 156 (43%) | 6 (2%) | 110 (30%) | 6 (2%) | 6 (2%) | 63 (17%) | 16 (4%) |
| Over-weight, n=108 | 58 (54%) | 1 (1%) | 27 (25%) | 3 (3%) | 0 (0%) | 15 (14%) | 4 (4%) |
| p-value^*^ | 0.010 | 0.741 | 0.431 | 0.629 | 0.530 | 0.539 | 0.051 |
| **Nutritional status for child (0 to 17 years), N=609** | |  |  |  |  |  |  |
| **Weight for Age Z score, n=556** |  |  |  |  |  |  |  |
| Normal, n=237 | 124 (52%) | 70 (30%) | 8 (3%) | 3 (1%) | 5 (2%) | 22 (9%) | 20 (8%) |
| Mild to Moderate underweight, n=274 | 132 (48%) | 60 (22%) | 24 (9%) | 2 (1%) | 9 (3%) | 34 (12%) | 23 (8%) |
| Severe underweight, n=45 | 17 (38%) | 11 (24%) | 11 (24%) | 0 (0%) | 0 (0%) | 2 (4%) | 6 (13%) |
| p-value^*^ | 0.188 | 0.143 | <0.001 | 0.782 | 0.549 | 0.232 | 0.507 |
| **Height for Age Z score, n=584** |  |  |  |  |  |  |  |
| Normal, n=325 | 166 (51%) | 84 (26%) | 19 (6%) | 1 (0%) | 9 (3%) | 36 (11%) | 27 (8%) |
| Mild to Moderate stunting, n=226 | 102 (45%) | 50 (22%) | 31 (14%) | 4 (2%) | 5 (2%) | 23 (10%) | 19 (8%) |
| Severe stunting, n=33 | 15 (45%) | 4 (12%) | 6 (18%) | 1 (3%) | 1 (3%) | 4 (12%) | 4 (12%) |
| p-value^*^ | 0.385 | 0.173 | 0.001 | 0.086 | 0.833 | 0.858 | 0.708 |
| **Weight for Height Z score, n=549** |  |  |  |  |  |  |  |
| Normal, n=258 | 134 (52%) | 74 (29%) | 7 (3%) | 4 (2%) | 5 (2%) | 24 (9%) | 25 (10%) |
| Wasting, n=274 | 132 (48%) | 59 (22%) | 30 (11%) | 1 (0%) | 9 (3%) | 28 (10%) | 24 (9%) |
| Over-weight or Obesity, n=17 | 6 (35%) | 5 (29%) | 4 (24%) | 0 (0%) | 0 (0%) | 5 (29%) | 0 (0%) |
| p-value^*^ | 0.344 | 0.150 | <0.001 | 0.32 | 0.629 | 0.050 | 0.537 |

^*^The Chi-squared test and Fisher's exact test were conducted as required.

**# Note:** Although our sample size was 1,404, we analyzed data based on the availability of weight and height measurements for both adults (n=600) and for nutritional status assessments among children aged 0 to 17 years (N=609), data were available as follows: Weight-for-Age Z score (n=556), Height-for-Age Z score (n=584), and Weight-for-Height Z score (n=549).
